# Unsettled hearing, responsible listening: encounters with voice after forced migration

**DOI:** 10.1515/applirev-2024-0088

**Published:** 2024-04-16

**Authors:** Katharina Brizić

**Affiliations:** 9174 University of Freiburg, Freiburg im Breisgau, Germany

**Keywords:** forced migration, voice, hearing, listening, ethics

## Abstract

In line with increasing forced migrations around the globe, there is also a growing need for ethical encounters between researchers and those forcefully displaced. This article focuses on responsible Listening, defined as the ethically motivated effort of researchers to conduct communication at eye level. However, findings have shown that listeners (e.g., researchers in institution or study settings) may already feel unsettled on the far more basic level of hearing a human voice, if certain implicit (e.g., aesthetic) expectations are not met. This makes encounters particularly vulnerable after forced migration where voices tend to be easily silenced. I will show by means of an empirical example what hearing a voice in its materiality, i.e. intonation, rhythm, accentuation etc., can set off, and how a privileged space of Listening to the Other can emerge.

## 1 Positioning as a listener

It was during the post-Stalinist period of the 1950s in former Yugoslavia that my father, right after school and military, experienced imprisonment and torture.1Cf., e.g., Grossmann (2021) for a literary account of the experiences of political prisoners at the camp at stake known as “Goli Otok”. In the 1960s, he fled to neighbouring Austria and attempted to cross the border three times until he succeeded and was recognized as a political refugee under the Geneva Convention. Of his flight he hardly ever spoke, and if he did, it was only in fragments, e.g. “We had to cross a little creek”, without much introduction or completion. Of his imprisonment and torture, however, he never spoke. Even later, during my childhood and adolescence, there was not a single word about it from my father’s side. I knew there was something unspeakable which for some reason was always there, and I knew the hard facts. Nevertheless, no feelings related to these experiences ever took explicit shape, not in any language of this multilingual, chatty family. Only once, in the 1980s – my sister and I had started crying loudly while watching an emotional children’s film at Christmas – only then, and totally unexpectedly, my father became aggressive like never before or after; and he stopped just as unexpectedly when we children, in a state of shock, stopped crying. It seemed as if this very sound had been unbearable for him: he could not stand hearing (our?) voices crying.

Towards the end of his life, more than half a century after his imprisonment and torture, all of a sudden he started talking about it, more precisely: crying. These occasions, however, were rare and strictly invariant: an initial sentence or phrase was regularly stifled by tears so that only four, five monotonous words could be heard before they were drowned in weeping. At this point, my responsibility set in, or more accurately, my rejection. I could not stand the sound. Notwithstanding the otherwise deep relation, I could not stand hearing him cry. I was entirely capable of hearing someone else crying – friends, aunts, uncles, grandparents, cousins, even strangers – and comfort them, but I was entirely incapable of fulfilling *his* need for comfort, of hearing *his* voice crying. What was yet even worse: I felt the need to protect and forcefully defend myself. Although I would have never voiced any of these feelings directly, I clearly expressed them by getting aggressively loud and busy in the attempt to bury that sound – for example, by suddenly tidying up the room, turning on the TV, refilling everyone’s glasses on the table and so forth. While my sister, my mother, all others gently *listened*,2In sharp contrast to myself, all others were completely silent, i.e. they were not producing any sound. This is why I perceived them as being *listening*. I was not even able to *hear*.

It is precisely this disturbing experience of the gap between listening and hearing that will form the centre of my contribution. In a time of increasing conflict and forced migration all around the globe, and the equally growing need for ethical encounters in research, I address “listening versus hearing” – beyond the personal experience – as an academic concern of vital importance. More concretely, my concern is whether or not the act of *listening to the human Other* can be impeded by the *incapability to stand hearing the Other’s voice* in all its disturbing, (un)aesthetic materiality; and if, consequently, *responsible listening* requires us to first of all *succumb to the very materiality of hearing*.

I will start to address this concern by outlining the four central theoretical concepts (Section 2) and the resulting empirical approach to the matter (Section 3); I will then proceed to demonstrating the approach on an empirical example (Section 4) and conclude with a discussion of the takeaway for applied (socio)linguistic research (Section 5). The frame and context, however, will always be the forcibly induced movement of people with the aim to escape conflict or persecution, among others (for an overview of definitions of forced migration, cf. Stanković et al. 2021). It is the transgenerational experience delineated above that gave rise to both this framing and my overall research focus on forced migration. In fact, that very experience shaped my childhood and young adulthood. This makes it all the more imperative to reflect on the hearing and listening practices of the researcher I am today, involved in ongoing encounters with voices after experiences of violence.

## 2 Approaching the cause theoretically

In sociolinguistics, the profound human need “to have one’s voice heard” and “develop a voice worth hearing” was already defined in Hymes’ (1996: 64) *Sociolinguistic concept of Voice* (henceforth ‘Sociolinguistic Voice’, capitalized in contrast to other, more material concepts of human voice). In this sense, Sociolinguistic Voice “…stands for the way in which people manage to make themselves understood or fail to do so” (Blommaert 2005: 4; Hymes 2003: 11). The concept comprises the full range of linguistic, artistic, behavioural, and other communication means, be they verbal or nonverbal, explicit or implicit, as well as the knowledge, competences, experiences etc. conveyed through communication. Moreover, along with success or failure, the concept also refers to the pressures and inequalities involved whenever the possibility to raise one’s Voice from an acknowledged position (Busch 2016: 321) is partly or fully at the mercy of others (Hymes 1996: xi; cf. already Spivak 1988 for the virtual impossibility of marginalized voices to be raised).

The position from which to speak is particularly vulnerable after forced migration, not only because of the new context one has arrived to, but equally – or even more so – due to the “old” context one has come from: when migration is preceded by profoundly threatening experiences, such as persecution, torture, sexual violence or genocidal threat, it is not uncommon for people – sometimes for a lifetime – to try keeping hidden what and how they had suffered (cf., e.g., Six-Hohenbalken 2018; Rosenthal 1995; Bar-On 1997; to name but a few). In this respect, not only one’s personal Voice (as a sociolinguistic concept) but also one’s voice in its basic, physical materiality (i.e., as an organ or instrument) remains mute. I refer to the latter as *material voice*, to underline the post-traumatic difficulty of already raising one’s voice on the material level of sound production, voice quality, intonation, and other sub-phonemic detail (cf., e.g., Hymes 1996: 61). In sum, it is well documented in research how often traumatic experiences remain unarticulated, and how often the “unsayable” prevails in discourse (cf. Rogers 2007). And yet, not all voices remain silent after experiences of violence. It has been reported both for adults and children that the materiality of “being loud”, too, can be an articulation of dealing with the past, be it consciously, e.g. when adults contest enduring discourses of violence (Sigona 2014), or subconsciously, e.g. when children fight against their own or their families’ experiences of war (Brizić 2022: 11; cf. also Bakhtin 1981 [1934–1935] for the potentially collective quality of an individual’s voice, which he refers to as polyphony).

As precarious as a voice after forced migration is also its “hearability” in discourse, as it requires “…listeners prepared to hear and understand…” (McNamara and Busch 2020: 330). For the listener, however, the “understanding” part is highly demanding and somewhat more difficult than just “hearing” – at least at first sight. Understanding is easily undermined by unequal power relations, e.g. in institutional settings, such as asylum procedures, education, or research contexts (Hymes 1996: 70 and 119; McNamara and Busch 2020: 330). In all these environments, it is regularly the applicant’s, student’s or interviewee’s voice that needs to convince, while the listener – the asylum authority, the teacher, the researcher – may attune to that voice or not, understand that voice in one way or the other, and so forth, also depending on the ideologies inherent to an institution or profession (cf. Bakhtin 1981 [1934–1935] for the concept of heteroglossia, i.e. the manifold ideologies connected to, and restricting, language use and its perception in different contexts). On this basis, and in line with the *Sociolinguistic Voice*, as mentioned above, I will define *responsible, i.e. ethically motivated, Listening* as the deliberate will and effort of the listener to communicate at eye level, and to become attuned to the Other’s Sociolinguistic Voice in its full range of knowledge and communication forms. Responsible Listening (henceforth capitalized in contrast to other concepts of listening) does not force the Other to be convincing3While the need to convince is rather typical for institutional settings, it is not usually followed in ethnographic approaches. However, ethnographic interviews are not immune to unequal power relations, so that an interviewee may still have the expectation that s*he will have to be convincing in order to be taken seriously. but rather leaves to the Other the authority to decide what should be understood by whom and how.

Apart from any understanding, however, it is the more basic hearing that seems to pose even greater difficulties for the listener, particularly within unequal, e.g. institutional, power relations. Although not nearly as well-studied as what I call *material voice*, it is exactly this *material, i.e. aesthetically motivated hearing* that tends to reveal the listener’s unfulfilled expectations and resulting disruptions. Primary school teachers in Austria, for example, often report that to their ears the voices of girls “from difficult backgrounds” sound “overly loud”, even “scolding”, and “wanting far too much” (Brizić 2022: 204–205; Brizić et al. 2021: 48–49). This, in turn, reveals the material-aesthetic expectations a teacher may have learnt to have as to how the voice of an immigrant girl “should sound”, e.g. soft, decent, modest, in sum: “adequate” for her “modest descent” (Brizić et al. 2021: 50; cf. Butler 2010 for the notion of frames of recognition which allow, or restrict, the capacity to apprehend the Other). The extent of this and similar clashes between voice and hearing becomes evident when we consider how important the full range of semiotic resources can be particularly after violent experiences: a voice’s materiality, e.g. its volume and sound, can become of vital relevance “…when ‘ordinary’ language is not ‘enough’” (McNamara and Busch 2020: 330; Rogers 2007: 92). In this spirit, a girl’s voice perceived as “loud” may be her primary means to express her wish to be heard; in the institutional context, though, the teacher’s ear learnt a certain materiality, an aesthetics which the “loud voice” does not meet. And in fact, institutional contexts are, not only in Austria, reported to give rise to students who, after forced migration, give up on voicing their experiences, competences, and needs (e.g., Thüne and Brizić 2022: 7–9). Similarly, yet much more frequently addressed in research, adults after forced displacement are reported to fail meeting institutional demands, e.g. when seeking medical care, and suffer “from having no admitted stance from whence to speak” (e.g., Busch 2016; cited from McNamara and Busch 2020: 328). Apparently, the institutional context can be so unsettled by the (un)aesthetics of hearing a voice that the ethics of Listening to the Other is out of reach.

Based on the findings outlined above, the following section presents an empirical approach to the core theoretical concepts at stake, i.e. *aesthetically motivated hearing* connected to *material voice*, and *ethically motivated Listening* associated with *Sociolinguistic Voice*. The larger frame of reference will continue to be forced migration.

## 3 Approaching the cause empirically

### 3.1 Framework

Fragments of a vessel which are to be glued together must match one another in the smallest details, although they need not be like one another. In the same way a translation, instead of resembling the meaning of the original, must lovingly and in detail incorporate the original’s mode of signification, thus making both the original and the translation recognizable as fragments of a greater language, just as fragments are part of a vessel. (Benjamin 2009 [1923]: 78; see German original in Benjamin 1972 [1923]: 18)

The philosopher Walter Benjamin, associated to Critical Theory and its protagonists in the Frankfurt School (cf. Maeße 2019), provides a metaphoric outline of what he sees as the core tasks of a translator: not the transmission of content or a “message”, but the *intense engagement* with the *materiality of the original*, i.e. its mode or structure (cf. Benjamin 1963 [1923]: 182–183 and 188). In other words, translation is seen as the (re)assembling of two structures: the original material – I would say: *voice* – on the one hand, and the attempt to trace it – I would say: *hear* it – on the other hand. In line with this approach, the original material or voice and the translation or hearing come together to form a new and larger structure, a “larger language” of its own. This potential to create a new entity makes the two parts uniquely interconnected. Acknowledging the necessity to focus on materiality, however, is exactly what makes Benjamin’s approach so fruitful to my own endeavour.

The same intellectual climate that influenced Benjamin’s take on translation, i.e. Critical Theory and the Frankfurt School, also lays the foundation for a discourse analysis that seeks to empirically understand the interplay between knowledge and power (cf. Maeße 2019: 289–291 and 299–300 for an overview, including conceptual differences between protagonists such as Bourdieu and Foucault). Once again, the empirical interest is not directed at content but at discursive *processes of interpretation* (cf. my concept of *hearing*), guided by the *exercise of power over materiality* (e.g., treating specific *voices* as “unaesthetic”, i.e. “illegitimate”). The material level, however, is also clearly transcended here: “voice” implies, for example, not only sound, but also knowledge, competence, experience, and so forth. This enables acts of assuming, or denying, the *responsibility of Listening* to someone, guided by the *exercise of power over knowledge* (e.g., treating particular *Sociolinguistic Voices* with their competences and experiences as “irrelevant”, “insufficient” etc. – in sum: as “illegitimate”; cf., e.g., Bourdieu 1986).

### 3.2 Approach

Within this discourse-analytical empirical framework, and grounded in the personal experience outlined in the beginning, I propose an empirical approach as committed to ethically motivated sociolinguistic research. For this aim, I will zoom in on an exemplary research context of hearing versus Listening to a human Other. However, I will not delve into discourse analysis. My approach will be somewhat “inverse”, as I will mainly focus on a particular role in discourse: that of the researcher as listener, together with her responsibility in analysing the account of a human Other, here: the narrative of a woman who experienced forced migration. In doing so, I ask: What does the researcher perceive while listening to the interviewee’s narrative? And how does this perception affect interpretation and understanding?

Yet, in contrast to existing critical reflections on interviews and the researcher’s perception of the Other (e.g., Kruse 2009), I will look at the researcher’s aesthetically motivated hearing of the Other. In line with this, I will focus on the materiality of voice, i.e. intonation, rhythm, accentuation, tempo and volume as enacted by the interviewee. I will ask: What difference does the material-aesthetic level make to the researcher’s ear? What is it exactly that attracts the researcher’s attention? What is its affective impact? What changes the researcher’s knowledge? And what does this mean in terms of power relations, i.e. the distribution of roles within the research context, and consequently also for the organization of knowledge with regard to interpretation? When analysing a narrative after forced migration, who lays the groundwork for whom? In sum, how does the *materiality of hearing* contribute to the researcher’s more conscious and responsible, i.e. *ethically motivated, Listening*? And how does the *materiality of voice* lead to a new perception of the Other’s knowledge, to a new perception of the Other’s *Sociolinguistic Voice*?

### 3.3 Research context

My approach will be applied to the example of a narrative-biographical account after forced migration. The data stems from a transnational, transdisciplinary study with 160 primary school children, their mothers, fathers, and teachers. The study was conducted among migrant communities in Vienna, Austria and Istanbul, Turkey. The aim was to understand transgenerational experiences of social, educational and linguistic marginalization, as well as resistance against social injustice, institutional inequity and linguistic discrimination (for details, see Brizić 2022: 17–52). The topics and aims had been communicated beforehand to the potential participants in order to enable informed consent (cf. AAA Committee on Ethics 2000).

The core part of the assessed data comprises 160 narrative-biographical interviews in total, conducted with the children’s mothers and/or fathers; almost all of them had experienced various forms of migration, including forced migration after politically or racially motivated violence. In a preformative understanding of social science (cf. Denzin 2001: 26), the guiding principle for the interviews was to provide a “stage” to the Other, i.e. rendering the interview “…a vehicle for producing performance texts (…) about self and society” (Denzin 2001: 24), with the interviewer representing the attentive, emphatic “audience” (Denzin 2001: 25).

Considering the roughly 20 languages spoken in the study sample, a large number of different interviewers was needed, with their involvement always depending on the interviewees’ language skills and choices; in sum, the languages chosen most frequently were Turkish and Kurdish.4In the migrant communities of Istanbul, Kurdish is among the languages with the most numerous speakers, while in Viennese migrant communities it is Turkish, followed by Kurdish (cf., e.g., Brizić and Hufnagl 2011, p. 25). As the project’s principal investigator, I trained the interviewers but was not involved in the conversations. My role was to subsequently listen to the recorded conversations in full length in order to overview and plan, or revise, further tasks (i.e., upcoming interviews; transcription; translation where needed; and narrative analysis, cf. Lucius-Hoene and Deppermann 2002). And it is exactly this process of my listening to the recordings – more precisely: my listening to one particular recording extract – which I will draw on in more detail.

### 3.4 Example

The extract I am referring to was, within the larger dataset, exceptional in several ways. Compared to the other interviews and interviewees, it was, first, significantly more difficult to follow this particular narrator, i.e. apprehend or “understand” her narrated experience and, hence, her Sociolinguistic Voice; second, it was considerably more difficult to apprehend or “stand” her material voice; and third, of all 160 conversations it was this biographical account that led to a disruption of the “known” and a subsequent shift in power relations within the research context. The account’s particularities, in turn, gave rise to its selection for a more detailed investigation.

The narrator, NAR, is a woman who was born in the 1980s in a Kurdish-speaking village in eastern Turkey. In her childhood she experienced forced migration after a large number of Kurdish villages had been accused of “collaborating with the PKK” and were, in consequence, destroyed by the Turkish military (cf., e.g., Bruinessen 1995). Due to the dangerous living conditions that led to forced migration, NAR had only little access to schooling. After displacement, NAR came to Istanbul where she still lives today, and where the interview takes place. As a woman from the economically disadvantaged east of Turkey and with her Kurdish family language excluded from the Turkish education system (cf., e.g., Coşkun et al. 2011), NAR is subject to manifold prejudice such as “lack of educational aspirations”, “suppression of women”, “traditionalism”, “separatism”, and many more (cf., e.g., Eppler and Benedikt 2017). Later on we may find out whether this context also motivated NAR’s choice to not speak her first language – Kurdish – in the interview but Turkish.

However, it was not NAR’s language choice but her tone of voice which unsettled me right from the beginning, when I started listening to the recording. And while it is the interviewer (henceforth INT) who conducted the conversation with NAR, it is always my own listening to the conversation that is central to the investigation. *I will, therefore, mark my personal comments as a listener in italics throughout the following section*, to maintain transparency while providing insight into my encounter with the materiality of NAR’s voice.

## 4 Hearing a voice

The conversation between interviewer INT and narrator NAR, conducted in Turkish, is one of the longest in the data corpus accumulated over my research years – *and perhaps also the most demanding one*, is my first thought when I start listening to the recording roughly a week after the conversation took place. *To my ears, NAR’s voice has a monotonous-indifferent yet also insisting, almost lurking tone* which leaves me highly confused, as it allows for at least two conflicting interpretations: a profound lack of interest in the conversation on the one hand, and a very specific, targeted expectation on the other. *But what, if anything at all, does NAR expect from the conversation?*

INT seems to perceive a similar orientation problem. Otherwise an experienced interviewer, she struggles to find common ground in the case at hand. This becomes apparent, for example, in her frequent questions to make sure she understood NAR’s elaborations correctly.

*For me – and maybe also for INT – the main problem is to better understand NAR’s family relations*. For NAR, however, her family relations seem to be closely connected to the research questions on migration, education, and language. This already becomes evident at the beginning of the interview when NAR’s Kurdish family language is at stake. Although the interviewer in her question referred to competence rather than origin, NAR puts special emphasis on rejecting the latter: “Our origin is not Kurdish. It is very close to Turkish.” And, with reference to how Kurdish came into the family at all: “The real Kurds are those from the side of my father.”

*My impression of a certain distance between NAR and her father*, still elusive at this point, gains further ground when NAR starts to recall how her father and his side of the family treated NAR’s mother: they incited NAR’s brother, still a child at that time, to hate the mother; and with only eight, nine years of age, NAR’s brother stalked his mother in the dark and threw stones at her. NAR’s father, however, was absent, leaving the mother without shelter against incitement, hatred, and stones. Even later, NAR’s brother did not overcome his strong aversion: “You are not my mother!”, NAR recalls one of her brother’s verbal attacks against the mother.

INT clearly has difficulties to follow. “Already as a child he did not like his mother?” and “Why did he not like her?” are the questions INT inserts emphatically during NAR’s portrayal of a history of assaults.

*Similar to INT, I am unsettled, too. This may be due to my confusion about what all this has to do with migration, language and education; or to my discomfort about the rigid male-female divide cutting through NAR’s family; or to NAR’s somehow expectant yet still highly monotonous tone of voice, standing in stark contrast to the violent events, with their reasons remaining opaque*. Even the explanation that NAR adds now – i.e., the fact that her little brother was frequently alone because the mother worked day and night to avert poverty – does not facilitate my understanding of the massive conflict.

*And there is another obstacle to my own, and potentially also to INT’s, comprehension: the frequent blurring of borders between NAR and her mother. This makes it difficult to distinguish between NAR speaking from her own point of view and NAR speaking from her mother’s*. The effect seems to be rooted, first, in NAR’s distribution of linguistic means (e.g., using the same pronouns for both her own and her mother’s perspective), and second, in her allocation of attention (i.e., allowing space only for her mother’s, never for her brother’s, thoughts, motives and intentions).

*And it may easily be these linguistic details that my listening attention is feeling triggered by, assuming imbalance and wondering: was there really nothing that the mother could have done to comfort the child, given his very young age? For how can a child hate his mother enough to throw stones at her?* – or, as the interviewer puts it with a slightly puzzled intonation: “HOW old was your brother then?”

For NAR, however, it seems to be dissatisfying that INT continues asking. This is evidenced by NAR’s answer providing the exact same information as already given before (cf. above): “He was about eight, nine years old.” Apparently, NAR sees no point in adding any defence for her brother’s behaviour. Rather, the mother’s perspective is seamlessly continued as NAR reports that her mother was left without information on the further whereabouts of the son.

Then, NAR takes a deep breath and continues: “My brother joined the military in Çanakkale, in the first, basic thing, in the unit.” With this, NAR fills the information gap regarding the whereabouts of the brother; apparently, he is now ten years older, old enough to be recruited to the Turkish military. However, NAR immediately shifts the focus back to her mother: “From Kars my mother went over to him, I assume: to attend the oath”.

*For me, the listener, this raises several new questions: Why does NAR’s mother, hated and uninformed for a full decade, now follow her grown-up son through Turkey, from the easternmost (Kars) to the westernmost (Çanakkale) border? How can the swearing-in ceremony, or the Turkish military as a whole, be that important to her? And is she not afraid her son might try to attack her again and her long journey might be in vain?* At the same time, I realize that my own and the interviewer’s perceptions are now going different paths: unlike me, INT shows no more signs of surprise nor does she ask any questions. *INT’s silence evokes my impression that she is now carefully listening without the need to interrupt.* But what is it that lets INT react so differently at this point although much remains unclear?

*And only now, made aware by INT’s silence, I can hear it as well: something has changed*.

From this point on, the conversation is provided in the transcript below, first in the Turkish original and then in the English translation, to make the following interpretation transparent, as it is always based on the original. Each line in the transcript represents an intonational unit, i.e., a phrase with a cohesive pitch contour (cf. Selting et al. 2011) as performed by NAR:

(1)

**Table:** 

Transcript: Turkish original									
NAR:	Narrator								
INT:	Interviewer								
Method:	Minimal transcript (cf. Selting et al. 2011)								
Signs used:	/	(basic rhythm unit)							
	AĞ	(accentuated syllables)							
	::	(strong lengthening of a sound)							
	↑	(strong pitch movement, either up ↑ or down ↓)							
Omitted:	Intonation, volume, tempo and breaks are described in detail								
	in the interpretation but omitted in the transcript,								
	in favour of making the overall structure visible.								
01	NAR:	**AĞ:**bim	/						
02		↑**AS**kere	/	**Gİ**tmişti		/	**ÇA**nakka-	/	**LE**
03		ilk acemi	/	şeyinde		/	birliğin-	/	**DE**
04		annem de	**KARS**tan onun yanına gidince herhalde yemin törenini						
05		oraya git-	/	miş yemin		/	töreni-	/	**NE**
06		↓sormuşlar	/	ha↑neyin		/	↓oluyo fi-	/	**LAN?**
07		**ANN:**em	/	↓demiş.		/			
08	INT:	hm::	/

(2)

**Table:** 

Transcript: English translation								
01	NAR:	my **BRO:**ther	/					
02		↑joined the **MIL**i-	/	**TA**ry	/	in **ÇA**-	/	nakka**LE**
03		in the first	/	basic thing			/	in the **UN**it^5^
04		from **KARS** my mother went over to him i assume to attend the oath						
05		she went there	/	to attend			/	the **OATH**
06		↓they asked	/	↑whose is ↓she			/	and so **FORTH**?^6^
07		my **MO:**ther	/	↓he said.			/	
08	INT:	hm::	/					

5 In order to make transparent and imitate the rhythm of the Turkish original, I will mark the rhythm units and accents in the English translation, too. However, a complete match with the original is not always possible or would not always make sense.

6 *Filan* can also be translated as *blah blah*, i.e. an onomatopoetic expression for a continued conversation. For the sake of clarity, however, I chose the more explicit translation *and so forth* [line 06].

Starting with “my BROther” [see line 01 above], NAR’s tone of voice, among others: her volume, has become remarkably different from before. *The words are so softly spoken and so distinctly embedded between ample breaks that I have enough freedom to guess what might be following after the break.* What follows, however, increases in volume, accelerating at once and displaying four clearly marked accents as if imitating a march in step: “…joined the MILi-TAry in ÇAnakka-LE” [line 02]. All of this – both the softness and the subsequent rhythmic accentuation – comes as a surprise, since NAR’s account had lacked any shifts in volume and accent so far. Moreover, the intonation units display a noticeable pattern: compared to line 01 (“my brother”), lines 02 and 03 are markedly longer, with the third line being accelerated to such a degree that there is time for only one final accent: “…in the first, basic thing, in the UNit” [03]. This tendency towards longer intonation units and faster speech reaches its peak in line 04, the longest and fastest so far, with the strong acceleration once again leaving time for just one single accent; the volume, however, is much softer now that NAR is speaking of her mother: “From KARS my mother went over to him, I assume: to attend the oath” [04]. This seems to have been a core information, as after that the lines grow shorter again: “She went there to attend the OATH” [05]. And while representing nothing but a repetition of content, line 05 still has an interesting rhythmic effect: it consists of the exact same number of syllables as does line 03 (cf. the Turkish original); moreover, line 05 also repeats the single-final-accent pattern from line 03. This stylistic back reference somewhat indicates a return to the point of departure – to the brother whose reaction is still not clear. Yet, by delaying this central issue, NAR intensifies the tension: she keeps the focus on her mother instead, and on the astonishment the mother causes at the military base, now that the soldiers finally have spotted her. The soldiers, obviously young men, are imitated by NAR with a youthfully high voice and a starkly rising, curiously asking intonation, as they wonder who the woman is who came from so far, and to which of the soldiers she belongs: “They asked: Whose {mother} is she, and so FORTH?” [06]. And only then, in line 07, NAR’s focus shifts back to her brother, the son who, as a child, had denied his mother. However, here and now, in an objectively reporting tone, he admits to all the world who she is: “My MOther, he said” [07].

*This is a strong, surprising outcome, its effect based on a wealth of musical aspects – as if this had been the first stanza of a poem*. The interviewer, too, acknowledges the performance by a soft and very long, expectant interjection [line 08; end of transcript].

*And in fact, what follows after a deep breathing pause confirms my first impression: to my ear, most stunningly, it is a poem, with its second stanza now being performed* (for a detailed transcript of the second stanza, albeit with a different analytical focus, cf. Brizić 2022: 110–115). However, this second part is solely about NAR’s mother, portrayed as truly exceptional: she is a woman from a village, born in the east of Turkey, deprived of education – and still, she made it all alone to the other end of Turkey, right to her son, despite his violence, and right into the military base, a core symbol of male power and the very centre of Turkey’s state power. NAR’s mother is a woman who dares, and she is a mother who cares: “He is my SON, she said”, as NAR concludes her second stanza, citing her mother for one last time with a moved and forgiving voice as she responds to the still curious soldiers.

The overall narrative effect, however, is based on artful, vocal characteristics shaping both stanzas alike. There is, first, NAR’s intonation making the “poem for the mother” (as I call it) different from everything before: the intonation is versatile and agile, moving back and forth between softer and louder volumes, the softest tones being reserved just for the mother, while imitation makes the other involved characters come to life. Second, there is rhythm, not only in imitation but also to build a structure of lines that grow longer and faster, peaking at the mother’s long journey, and then growing slower and shorter again. The third and strongest effect, however, seems to be rooted in each stanza’s beginning and end: in the first stanza, the initial line presents NAR’s brother (“My BROther”), to be asked by his fellow soldiers, while the final line quotes him confessing his mother (“My MOther, he said”); in the second stanza, the initial line presents NAR’s mother (“My MOther”), to be approached by the soldiers, while the final line cites her confessing her son (“He is my SON, she said”). Recited with a highly rising, then gently falling intonation, it is the story of a mother forgiving her son. NAR’s poem is dedicated solely to her, with the most eye- and ear-catching particularities being NAR’s voice circling around her mother, and INT’s appreciative listening throughout.

*Then NAR returns to the monotonous tone from before*. She reports not only her mother’s but also her own life as torn between male violence and female forgiveness, male aversion against, and female love for, education. It was, for example, NAR’s mother who insisted on her school enrolment, while NAR’s male relatives disapproved. Then there is the experience of discrimination and exclusion as being marked “Kurdish” both in everyday life and at the workplace. NAR’s reaction is to dissociate herself from “being Kurdish”, which she experienced as threatening; at the same time, she distances herself from anything “male” which she experienced as violent, thus establishing an essentializing connection between the attributes “male”, “Kurdish”, and “threatening”. Her resulting strategy against both discrimination and violence is her positioning as an “education-affine, Turkish-speaking female”, a woman determined to resist male force, closely oriented towards her mother, the conqueror of foreign worlds who rescued her, supported her, and paved her way.

*And while NAR’s monotonous intonation prevails for the rest of the interview, the insisting, expectant impression from the beginning has faded*. It seems that INT’s affirmative, deeply moved listening to the poem for the mother fulfilled NAR’s expectations. In this way, NAR’s poem also reveals a larger structure: for her, the whole conversation may have served the profound need to agree on what is good, human and may rescue from anything evil; and this may precisely be the reason why it was of vital importance for NAR to receive INT’s embracing response. In fact, affirmative listening may have been so vital for NAR that she performed her poem solely to this end.

## 5 Unsettled hearing, responsible listening. Discussing aesthetic and ethical dimensions

What was it that attracted my attention, when I, as the listening researcher, was engaged in the interview recording? What interrupted the process and gave an impetus for change? And what was the outcome?

The interview between the two female conversation partners, NAR and INT, took place in a context of forced migration, which had, prior to the interviews, also been agreed upon with the interviewees to be one of the main conversation topics. My attention was consequently directed towards forced migration and its – potentially – traumatic impact.

When I started listening to the interview recording roughly a week after the conversation, I realized that NAR’s way of narrating in fact showed potential characteristics of traumatization. Above all, she seemed to boil down various threatening contexts (forced migration, educational deprivation, linguistic discrimination) to one single, all-encompassing story line (i.e., male violence within her family). In research this is known to be a coping strategy not uncommon in the accounts of traumatized persons (cf., e.g., Rosenthal 2003). In sum, it was my conviction at this point that I was *Listening to a Sociolinguistic Voice* and its potentially traumatizing experiences of forced migration.

Another potential indication of trauma was, to my ear, NAR’s highly monotonous intonation despite reporting dramatic events (cf. again Rosenthal 2003). And while this seemed not too surprising, it was still an aspect of NAR’s *material voice* that was hard for me to stand, i.e. *hear*. Moreover, and although I was in fact hearing a conversation I had never been involved in, my unsettled hearing felt further triggered by another perception: I recognized an intonation of insistent asking at the end of numerous of NAR’s statements. It was as if NAR was expecting something from the conversation, i.e. from the interviewer and – as I remember to have felt – also from me, the researcher. My reaction was an overwhelming sense of reluctance. Did I fear I would not be able to meet whatever expectations of a woman I had never met personally? And was the interviewer similarly unsettled, given INT’s frequent questions to make sure she had understood everything? In light of the fact that I had trained INT, it did not come as a surprise that INT’s and my reactions seemed to be similar in many respects.

The interruption of the process happened roughly 13 min after the conversation had started, and to my ears it happened without prior notice or introduction: at once, NAR’s *voice* lost the monotonous overall intonation as well as, at the end of the sentences, the insistingly-asking tone. It was an interruption in form of an aesthetic move, i.e. a change in NAR’s *material voice*, which I noticed as a change in the *materiality of my hearing*, and it abruptly put an end to my sense of reluctance. More precisely, it was, first of all, the interviewer who attracted my attention to the change, simply because INT stopping asking questions. And only then I noticed that there must have been a move; and only then I noticed the move itself: NAR’s voice had – to my ear – begun to “recite” or “sing”.

This change went much further. What was perceived as a move in the Other’s voice caused a profound shift in my own, the listening researcher’s, knowledge. In the course of what I began to understand as a poem or song, I also understood that I had up to now only *thought* to be Listening to Sociolinguistic Voice. Yet, far from the presumed, I had been involved in aesthetically motivated hearing, going even as far as “judging” on the (un)aesthetics of what I heard; and I had been expectantly waiting for “content”, quite in contrast to what the analysis of a narrative, of a discourse is all about (cf. Section 3). There had been no Listening by the researcher whatsoever, only listening without ethical motivation, and an aesthetic hearing only in the sense of wishful thinking, oriented on (familial?) experiences of what is hearable or not, endurable or not, painless or not. In all-encompassing immodesty, I had tried to avoid pain.

The disruption of the “known” came as a shift in power, too, as the roles considerably changed: neither me, the researcher, nor the interviewer I had trained had been providing a “stage” to the Other (cf. Section 3), be it directly in face-to-face communication or indirectly while listening to the recording. Rather, it was NAR, the Other, who started providing a stage *for us*, rendering the interview a vehicle for the listeners’ self-performance, in my case: for avoiding old pain. And NAR, the interviewee, was an emphatic audience indeed. Whether consciously or not, she must have understood that the listeners – INT, and thereby I, too – were in need of something else; NAR’s expectant intonation, for example, had not led anywhere. And so she prepared the ground *for us* – INT and me – by changing her strategy from “expectantly asking” to “provoking the desired reaction”. And she did so by a compelling performance: a song. It was her voice’s capacity to slide into my attention so I would not have to move myself. It was her voice’s capacity to create poetry in the listening researcher’s ear.

In this way uniquely addressed by the Other (Biesta 2016), I realized that I had until now *avoided* to encounter NAR’s Sociolinguistic Voice. This, however, was also the chance to abandon that aim for the moment, for there seemed to be something more important to be done: to engage, without reservation, in *aesthetically motivated hearing*. I had been doing so anyway – but now I was aware of it, and had been invited to it by NAR; why not accept NAR’s invitation and indulge? With my attention now fully directed towards the Other’s *materiality of voice*, I could sensually feel my becoming attuned to it through an intense engagement with tone and rhythm, intonation and accentuation. This was still done without “knowing” where the path would lead, but with a new and conscious “exercise of power over materiality”, i.e. respect for the uncertainty and provisionality of any material hearing (cf. Benjamin 2009 [1923]: 75).

What was it, after all, that NAR had invited us to hear? We will never know, and probably we need not even try. The only answer I can give as a listener is that NAR’s account was music *to my ears*. What results from this perception is no less significant, albeit nowhere communicated in explicit words, i.e. “ordinary language” (cf. Section 2). Rather, it was almost exclusively organized through voice, with all of the material means circling around, and appreciating, a female family member: NAR’s mother.

From there, only a small step was left for the listening researcher to take. The mother was harassed – by men; the mother and/or the daughter, NAR, was injured – by men. The mother and the daughter were hindered from schooling – by men. And the mother and the daughter would have been alone if they had not had each other. At some points during the conversation, it was not even possible for the listener to decide who was what, and it may well have been irrelevant since NAR waved clear distinctions between herself and her mother. However, to be believed, appreciated and embraced by the listener – merely this seemed to have been important to NAR. But what about the trauma that sounded so evident in her voice? It was, as far as we can know, clearly not about forced migration. If there was, or is, trauma, then most likely due to gender-based violence, i.e. physical or other force against women and girls (cf., e.g., Altınay and Arat 2009; Çağlayan 2008). In consequence, if any self-definition became apparent in NAR’s account, then most evidently that as a female, and as utterly determined to resist, and overcome, forceful male dominance.

How far must my expectation – i.e., politically or racially motivated violence – have been from what NAR wanted to communicate, i.e. gendered experiences of violence, female perspectives on trauma and desire, in short: *her* experience, *her* expectation regarding the conversation. This expectation was hardly about the listener “understanding a content”, nor about “emphatic questions”; rather, it was about affirmative Listening, a silent embrace at least. And still, I was so engaged in avoiding the collective stereotype of “suppressed women” (cf. Section 3) that the Other’s right to individuality and uniqueness, together with her signalling a need, remained long without an ethical answer. It was only via her material voice, after all, that her *Sociolinguistic Voice*, her power over knowledge, could take shape in the encounter. With her poem for the mother, NAR ensured that I, the researcher, finally heard the call. Only then could I, the listener – and with it a radically different biographical pain – become *consciously* involved in hearing, in not expecting any more, not knowing any more, in wondering, enjoying, getting lost, embracing, in short: *ethically motivated Listening – beyond the boundaries of what is shared*.

## 6 Proposing some implications for applied (socio)linguistics

In applied linguistic and sociolinguistic research, one must never think anything is shared. The insider-outsider dilemma, however, is not new (cf., e.g., Liu and Burnett 2022), yet the materiality of voice can tackle it from a different angle: it seems that the intense engagement with the material voice of the Other helps to make more radical decisions on what one “knows” or “oneself has experienced”, and what not. The materiality of voice helps unmasking what we, as researchers, expect; what we hear; what we believe to hear; what our hearing denies, likes, hates, and why.

The example in Section 4 may have illustrated how dangerous it is to not be aware of one’s own hearing. In the case at stake, this unawareness may well have caused NAR’s performance of the poem for the mother, likely intended to “help” the listener, i.e. provide the best possible conditions to ensure that the listener starts hearing, and, finally, Listening. The poem is certainly a “key story” of the interview, connected to a masterly performance (cf. Brizić 2022: 195) with principles of organization that are also found in other contexts of orality worldwide (see Hymes 1996). But we do not know what it takes for a potentially traumatized person to leave her monotonous tone behind, only to ensure hearing – since NAR returned to the monotonous intonation right after the poem. I consider this an important incentive to ask: What would it take to enable encounters *without* forcing the Other, albeit unconsciously, to “help”, even if this “helping” is beautiful?

One opportunity to ensure “hearing without forcing” is often provided right at the beginning of an interview. In her early ground-breaking work on the Shoa, Rosenthal (1995: 154), for example, already gave accounts of how interviewees actually “announce” the conditions they need for their biographical narration. The materiality of human voice, however, is still to be included in this field of research. One of the core suggestions emerging from my approach is, therefore, a far more sensual understanding of the ethical demands in encounters with voices after forced migration and other violence. This would not only honour contexts of orality where voice is masterfully employed, and not only support the Sociolinguistic Voice to emerge, but it would encourage a privileged path for the researcher and the Other towards developing their difference into a joint “larger language”, a rare space in our violent times, where uniqueness and responsibility for the Other are no antagonisms.
